# Two sample Mendelian Randomisation using an outcome from a multilevel model of disease progression

**DOI:** 10.1007/s10654-023-01093-2

**Published:** 2024-01-28

**Authors:** Michael Lawton, Yoav Ben-Shlomo, Apostolos Gkatzionis, Michele T. Hu, Donald Grosset, Kate Tilling

**Affiliations:** 1 Population Health Sciences, Bristol Medical School, University of Bristol, Bristol, UK; 2 MRC Integrative Epidemiology Unit, University of Bristol, Bristol, UK; 3 Nuffield Department of Clinical Neurosciences, Oxford University and Oxford University Hospitals NHS Foundation Trust, Oxford, UK; 4 School of Neuroscience and Psychology, University of Glasgow, Glasgow, UK

**Keywords:** Two sample Mendelian Randomisation, Parkinson’s disease, Multivariate meta-analysis

## Abstract

Identifying factors that are causes of disease progression, especially in neurodegenerative diseases, is of considerable interest. Disease progression can be described as a trajectory of outcome over time—for example, a linear trajectory having both an intercept (severity at time zero) and a slope (rate of change). A technique for identifying causal relationships between one exposure and one outcome in observational data whilst avoiding bias due to confounding is two sample Mendelian Randomisation (2SMR). We consider a multivariate approach to 2SMR using a multilevel model for disease progression to estimate the causal effect an exposure has on the intercept and slope. We carry out a simulation study comparing a naïve univariate 2SMR approach to a multivariate 2SMR approach with one exposure that effects both the intercept and slope of an outcome that changes linearly with time since diagnosis. The simulation study results, across six different scenarios, for both approaches were similar with no evidence against a non-zero bias and appropriate coverage of the 95% confidence intervals (for intercept 93.4–96.2% and the slope 94.5–96.0%). The multivariate approach gives a better joint coverage of both the intercept and slope effects. We also apply our method to two Parkinson’s cohorts to examine the effect body mass index has on disease progression. There was no strong evidence that BMI affects disease progression, however the confidence intervals for both intercept and slope were wide.

**Supplementary Information:**

The online version contains supplementary material available at 10.1007/s10654-023-01093-2.

## Introduction

Determining causality in observational cohort studies can be difficult due to problems with both measured and unmeasured confounding. Two sample Mendelian Randomisation (2SMR) is a technique used to determine causal relationships in observational studies that leverages genetic data as instrumental variables. Mendel’s laws state that genes are randomly assigned at conception hence they are ideal candidates for instrumental variables. Under the three instrumental variable assumptions 2SMR allows us to determine the causal relationship between an exposure and an outcome that is unaffected by confounding and reverse causation. This technique has gained popularity in recent years with the number of publications per year growing rapidly [1] and has mostly been used to determine relationships where the outcome is developing a disease (i.e. a binary outcome using logistic regression) [2, 3] or a health marker such as blood pressure (i.e. a continuous outcome using linear regression) [4–6]. Some research has also been carried out where the outcome is time to event [7], but to our knowledge not where the outcome is the trajectory of disease progression over time. We are interested in causal inference where the outcome is a repeatedly measured trait in individuals with a particular condition.

Neurodegenerative diseases like Parkinson’s disease (PD) and multiple sclerosis (MS) lead to disability that typically worsens over time. Identifying factors that are related to disease progression could lead to developing new treatments and better counselling of patients at diagnosis. Disease progression in observational cohorts has been studied before in both PD [8] and MS [9] using multilevel models (also called growth models, repeated measures models and random slope and intercept models). In these circumstances we are interested in the trajectory of some continuous trait over time which in the case of PD and MS is usually related to the severity of motor disability. When working with a multilevel model we are often interested in both the intercept (disability at a time of zero) and the slope (rate of change in disability over time) and it is important to study the effect of the exposure on both the intercept and slope.

An important issue when carrying out 2SMR in a cohort of individuals who have a disease is the index event bias phenomenon. The bias occurs when the MR instrument/exposure affects disease incidence and there are confounders of incidence and progression. Incidence is then a collider (common effect) of the MR instrument/exposure and the confounders, and restricting to a sample of diseased individuals will induce spurious associations between these variables, biasing the MR analysis [10]. This “index event bias” can affect causes of the disease—risk factors for disease progression which are not causes of disease (including treatment) do not suffer this bias.

The index event bias phenomenon has received attention for both GWAS and MR studies [10, 11]. Two methods to address this bias are Dudbridge et al.’s index event bias correction [11] and Slope-Hunter [12] which were both originally developed for genome wide association studies and would also require additional data from a genome-wide association study of developing the disease in question. Methods for addressing index event bias have been reviewed by Mitchell et al. [13] and another method has been developed for index event bias within MR studies [14]. We do not address index event bias in the methodology we develop here buts its implication for our applied example is discussed.

This article presents a simulation study for a multivariate method to carry out 2SMR where we are interested in the causal effect an exposure that does not vary over time on both the intercept and slope in a model of disease progression. This method uses multivariate meta-analysis which is often used in meta-analysis of diagnostic studies when researchers are interested in both the sensitivity and specificity of a test [15, 16]. Our aim is to examine bias and coverage of both separate and joint confidence intervals using this approach. We also apply this method to two cohorts of individuals with PD where we are interested in the causal effect of body mass index (BMI) on severity at diagnosis (intercept) as well as disease progression (slope) using motor symptom severity measured by the Movement Disorder Society Unified Parkinson’s Disease Rating Scale (MDS-UPDRS) [17]. BMI has been shown previously to be related to disease severity [18] and progression [19] in PD and an MR study has shown that lower BMI was associated with higher risk of developing PD [2].

## Methods

### Two sample Mendelian Randomisation

2SMR is a technique to estimate the causal effect ($\alpha$) of an exposure on some outcome (y) using genetic data, usually single nucleotide polymorphisms (SNPs), as instrumental variables. The effect of each SNP on the exposure (${\widehat{\gamma }}_{k}$ for the kth SNP) can be obtained from a genome-wide association study (GWAS). In the case of 2SMR the SNPs used should be independent. The effect of the SNPs on the outcome would come from a completely separate sample, hence the name two sample Mendelian Randomisation (MR), for example they might come from some regression model with the following format.

1 $$f(y_i ) = \beta_0 + \beta_{1k} \cdot G_{ik}$$

where ${G}_{ik}=$ The number of effect alleles (0, 1 or 2) for the *i*th individual and *k*th SNP, ${y}_{i}$= Outcome for the *i*th individual.

Using the data from these two samples the causal effect can be estimated from a weighted regression of the estimated effects of SNPs on the outcome (${\widehat{\beta }}_{1k}$) against the estimated effects of SNPs on the exposure (${\widehat{\gamma }}_{k}$). This is weighted by the inverse of the variance of the effects of SNPs on the outcome ($var({\widehat{\beta }}_{1k})$).

2 $$\hat{\beta }_{1k} = \alpha \cdot \hat{\gamma }_k ,{\text{weighted}}\,{\text{by }}\frac{1}{{var(\hat{\beta }_{1k} )}}$$

This can also be thought of as a meta-regression or a meta-analysis of Wald ratios (in this example${\widehat{\beta }}_{1k}/{\widehat{\gamma }}_{k}$) which are all identical mathematically. There are other approaches to 2SMR which relax the assumptions in some way the most common being the MR-Egger [20], Median [21], and Mode [22] approaches. In one-sample MR analyses correct specification of the exposure model is not a requirement. For two-sample approaches (the focus of this article) if the two populations are homogenous including the sampling distributions of the instruments (in this case SNPs) then two-sample MR is asymptotically unbiased even if the exposure model is not correctly specified. If the two samples are heterogeneous then further assumptions are required, e.g. that the SNP-exposure model is linear and correctly parameterised [23].

### Disease progression models

We are focussing here on multilevel models (also called growth models, random slope and intercept models and hierarchical models). They are often used to model outcomes across time where repeated measurements are available and can easily accommodate unbalanced data (number of observations per individual differs and the time between observations is not constant). They account for the non-independent nature of repeated measurements within an individual by incorporating random effects into a standard regression model. A simple model with no covariates other than time where the relationship with time was linear would have the format

3 $$y_{ij} = \beta_0 + u_{0i} + \left( {\beta_1 + u_{1i} } \right) \cdot t_{ij} + \varepsilon_{ij}$$

*i* = 1, …, *n* (number of individuals), *j* = 1, …,*n*_*j*_ (number of observations per person), *t*_*ij*_ = time at the *j*th time point for the *i*th individual, *y*_*ij*_ = outcome at the *j*th time point for the *i*th individual, ${\varepsilon }_{ij}\sim N[0,{\sigma }^{2}]$—this is the residual variation, also sometimes called level 1 variation in context of multilevel models, assumed to be normally distributed, $$\left[ {\begin{array}{*{20}c} {u_{0i} } \\ {u_{1i} } \\ \end{array} } \right]\ \sim N\left( {0,\left[ {\begin{array}{*{20}c} {\sigma_0^2 } & {\sigma_{01} } \\ {\sigma_{01} } & {\sigma_1^2 } \\ \end{array} } \right]} \right)$$—these are the patient level random effects, assumed to be bivariate normally distributed.

**Fig. 1:**
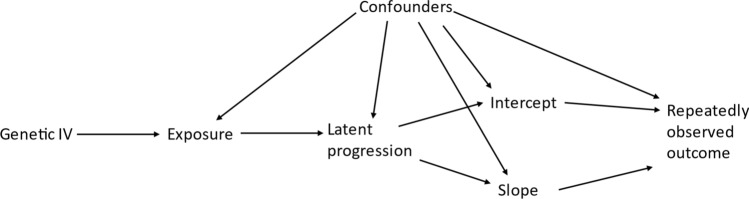
Assumed Directed Acyclic Graph for the effect of exposure on disease progression

For simplicity in this paper we are assuming models with a continuous outcome, only linear time, no complex level 1 variation, and no informative drop-out however these models could easily be adapted as explained later in the discussion. Within this paper we are assuming a particular directed acyclic graph (DAG), see Fig. 1. That is the intercept does not cause the slope but the exposure causes some latent progression trait that causes both the intercept and slope.

In the context of causal effect modelling and the DAG we would be interested in the effect that some exposure had on both the intercept and the slope. In this manuscript, we will assume that the value of the exposure is fixed for each individual and does not change over time. If the exposure had an effect on the intercept but not the slope then we could say that the exposure was related to disease severity at baseline and if there was an effect on the slope that the exposure was related to disease progression (or rate of change). If there was a single confounder of the effect the exposure has on the intercept and slope we would have the following longitudinal model

4 $$y_{ij} = \alpha_0 + u_{0i} + \alpha_1 \cdot X_i + \alpha_2 \cdot C_i + \left( {\alpha_3 + u_{1i} + \alpha_4 \cdot X_i + \alpha_5 \cdot C_i } \right) \cdot t_{ij} + \varepsilon_{ij}$$

*X*_*i*_ = the exposure for the *i*th individual, *C*_*i*_ = the confounder for the *i*th individual.

Here ${\alpha }_{1}$ is the causal effect of the exposure on the intercept and ${\alpha }_{4}$ the causal effect of the exposure on the slope.

In the context of 2SMR we would then be fitting a multilevel model for each SNP such that

5 $$y_{ij} = \beta_{0k} + u_{0i} + \beta_{1k} \cdot G_{ik} + \left( {\beta_{2k} + u_{1i} + \beta_{3k} \cdot G_{ik} } \right) \cdot t_{ij} + \varepsilon_{ij}$$

and from the GWAS study we would know (in the case of a continuous exposure with linear regression) that for *k* SNPs

6 $$X_i = \gamma_{0k} + \gamma_{1k} \cdot G_{ik} + \varepsilon_i$$

If we insert Eq. 6 into Eq. 4 and compare the with Eq. 5 we can see that

$$\alpha_1 \cdot \gamma_{1k} = \beta_{1k} \,{\text{and}}\,\alpha_4 \cdot \gamma_{1k} = \beta_{3k}$$

A naïve approach to 2SMR would be to carry out a meta-regression of the ${\widehat{\beta }}_{1k}$’s and ${\widehat{\gamma }}_{1k}$’s to estimate ${\alpha }_{1}$ the causal effect of the exposure on the intercept. In a standard approach to 2SMR this would be a fixed-effects meta-regression weighted by the inverse of the variance of the ${\widehat{\beta }}_{1k}$’s. Under the assumption that these SNPs are independent then meta-regression would be a valid method to estimate ${\alpha }_{1}.$

Then we could carry out a separate meta-regression of the ${\widehat{\beta }}_{3k}$’s and ${\widehat{\gamma }}_{1k}$’s to estimate ${\alpha }_{4}$ the causal effect of the exposure on the slope. Again this would be a fixed-effects meta-regression weighted by the inverse of the variance of the ${\widehat{\beta }}_{3k}$’s.

This would be complicated by each SNP having an effect on both the intercept and the slope and those estimates could be correlated. Hence there is a covariance between the ${\widehat{\beta }}_{1k}$’s and the ${\widehat{\beta }}_{3k}$’s whilst ${\alpha }_{1}$ and ${\alpha }_{4}$ could also be correlated. Using multivariate meta-regression [24] we could estimate both ${\alpha }_{1}$ and ${\alpha }_{4}$ jointly incorporating the covariance between the ${\widehat{\beta }}_{1k}$’s and the ${\widehat{\beta }}_{3k}$’s. Multivariate meta-analysis and meta-regression is a likelihood based method that applies weights to the likelihood using the covariance matrix of the ${\widehat{\beta }}_{1k}$’s and the ${\widehat{\beta }}_{3k}$’s. To be consistent with standard 2SMR we have used a fixed effects estimation. Doing the estimation jointly allows us to estimate not only the effect an exposure has on the intercept and on the slope (along with the standard errors) but also the covariance between these two estimates.

### Application of approach to Parkinson’s cohort

We will motivate this 2SMR approach with a real-data application, looking at the causal effect of body mass index (BMI) on PD. For our application, we will use 97 SNPs from the Locke et al. GWAS [25] as genetic instruments for BMI. For PD, we will use two parallel cohorts, namely the Oxford Discovery cohort and the Tracking Parkinson’s cohort [26, 27]. At recruitment individuals had to be within 3.5 years of diagnosis and are followed up every 18 months in both studies. The Oxford Discovery cohort was recruited from 11 hospitals in the Thames Valley Region between 2010 and 2016. The Tracking cohort was recruited from 72 sites across the UK between 2012 and 2014. We have previously carried out a genome-wide association study of motor and cognitive progression in these two cohorts [28]. Our analysis samples will be restricted to those with a probability of diagnosis of PD ≥ 90% as rated by a neurologist at the latest available visit. This is an attempt to exclude individuals who were incorrectly diagnosed with PD as it has been shown previously that some individuals diagnosed with PD will turn out to have another disorder [29, 30]. More details about the individuals are included in the results. Informed consent was obtained from all individual participants included in both studies.

The outcome (MDS-UPDRS part III) we are using consists of 33 questions rated on a scale of 0–4 giving a total score that ranges from 0 to 132 [17]. This is the most common instrument for motor symptom severity within the field of PD and is often used as the primary outcome in RCTs. For people with PD it does not exhibit a floor or ceiling effect and although technically measured at an ordinal scale, its range is large enough that it can be considered approximately continuous. We are using time since diagnosis as the time axis in our multilevel models.

### Simulation study

For our simulation study we have used the ADEMP (aim, data, estimands, methods, performance) guidelines to inform the design and reporting [31].

***Aim*** The aim of this study was to investigate different methods for two sample Mendelian Randomisation where the outcome is a multilevel model of disease progression.

***Data generating mechanism*** We simulated data for 10,000 individuals. Our simulation was partially informed by the real-data application studying the effects of BMI on Parkinson’s disease. We generated genetic data based on the Locke BMI GWAS paper [25] which reported on 97 GWAS hits. The number of effect alleles for each individual was simulated from a binomial distribution with n = 2 and p = effect allele frequency from the BMI GWAS paper. Using the beta-coefficients reported from the BMI GWAS paper we were able to create an exposure measurement for each individual by multiplying the simulated SNPs by their beta-coefficients (${\widehat{\gamma }}_{k})$ and then adding on an additional residual variance term. In this example we have simulated a single exposure for each individual that does not vary over time. We also simulated a continuous confounder (for the exposure and outcome) that would describe 50% of this residual variance in the exposure. This variance term and confounder were simulated for both an R^2^ of 2% and 10% to assess whether the variance explained by our genetic instruments affected the methods performance. To construct this variance term we calculated the expected variance of the 97 SNPs by the sum of ${\widehat{\gamma }}_{k}$^2^*2*p*(1 − p). Note that in the BMI GWAS paper the SNPs actually explained 2.7% of the variance of BMI.

We then simulated balanced longitudinal data with 7 visits per person observed at times of 0, 1, … to 6. The data was simulated under six different scenarios described below.

After simulating the SNPs, the exposure and the confounder the longitudinal data was simulated under the following model with the same definitions as in Eq. 4 above.

7 $$y_{ij} = \alpha_0 + u_{0i} + \alpha_1 \cdot X_i + \alpha_2 \cdot C_i + \left( {\alpha_3 + u_{1i} + \alpha_4 \cdot X_i + \alpha_5 \cdot C_i } \right) \cdot t_{ij} + \varepsilon_{ij}$$

***Fixed effects***

In scenario 1 we used the following parameter values.

$$\alpha_0 = 24,\alpha_1 = 2,\alpha_2 = 1,\alpha_3 = 2.25,\alpha_4 = 0.45,\alpha_5 = 0.225$$

In scenario 2 we altered the effect the exposure and confounder had on the slope to change the relative effect of intercept vs. slope, such that

$$\alpha_4 = 0.25,\alpha_5 = 0.125$$

In scenario 3 we altered the effect the exposure had on the slope to be zero

$$\alpha_4 = 0$$

Finally, in scenario 4 we altered the effect the exposure had on the intercept to be zero

$$\alpha_1 = 0$$

Scenarios 5 and 6 used the same fixed effects as scenario 1.

The effect the exposure and confounder had on the intercept (${\alpha }_{1},{\alpha }_{2}$) and the slope (${\alpha }_{4},{\alpha }_{5})$ was based on the expected s.d. of the exposure and confounder. This was to ensure similarity of effect sizes when R^2^ was 2% and 10% since changing the variance term when simulating the exposure would also alter the variance of the exposure. Note the direct effect the confounder has on the intercept and slope was 50% of the total effect the exposure had (except in scenarios 3 and 4 where the confounder slope and intercept effects are zero), however there is also a substantial indirect effect as the confounder described 50% of the residual variance in the exposure.

***Random effects and residuals***

For scenarios 1, 2, 3 and 4 the patient level random effects were simulated from

$$\left[ {\begin{array}{*{20}c} {u_{0i} } \\ {u_{1i} } \\ \end{array} } \right] \sim N\left( {0,\left[ {\begin{array}{*{20}c} {88.9} & { - 6.0} \\ { - 6.0} & {4.6} \\ \end{array} } \right]} \right)$$

In the fifth and sixth scenarios we altered the covariance of the random effects thus allowing us to alter the estimated covariance between the SNP-intercept and SNP-slope effects. In scenario 5 the covariance was adjusted to be positive (6.0) and in scenario 6 the covariance was adjusted to be twice as large as scenario 5 (12.0). The level 1 residuals were simulated from

$$\varepsilon_{ij} \sim N(0,{51}.{9}).$$

The random effects distribution (for scenarios 1, 2, 3 and 4) and the level 1 residual term were taken from an analysis on the PD Discovery cohort MDS-UPDRS III data (see “Application of approach to Parkinson’s cohort” section above).

Finally we carried out a sensitivity analysis with a smaller sample size and unbalanced data with fewer observations. This used the same approach as scenario 1 with an R^2^ of 10% but only 1,000 individuals (instead of 10,000) and unbalanced data. To create the unbalanced data each individual had 1 observation with 20% probability, 2 observations with 30% probability, 3 observations with 30% probability or 4 with 20% probability. The baseline time was simulated from a uniform distribution between 0 and 3.5 and the time between each observation was simulated from a uniform distribution between 1.4 and 1.6.

For each scenario, 1,000 simulations were run. A brief description of the scenarios is given below:

1. Baseline simulation
2. Reduced effect on slope
3. No effect on slope
4. No effect on intercept
5. Positive covariance within people
6. Large positive covariance within people

*Estimands/targets of analysis* The estimand of interest is the effect the exposure (BMI) has on the intercept (${\alpha }_{1}$) and also the slope (${\alpha }_{4}$) in the model of disease progression (Eq. 4).

***Methods:***

For each SNP we fit a multilevel model of the following format

8 $$y_{ij} = \beta_{0k} + u_{0i} + \beta_{1k} \cdot G_{ik} + \left( {\beta_{2k} + u_{1i} + \beta_{3k} \cdot G_{ik} } \right) \cdot t_{ij} + \varepsilon_{ij}$$

All other variables are as described above in “Disease progression models” section.

After fitting one multilevel for each SNP we considered two different approaches. The first is a naïve approach doing two separate 2SMR’s as described above in “Disease progression models” section one for the intercept (using ${\widehat{\beta }}_{1k}{\prime}s$) the other for the slope (using ${\widehat{\beta }}_{3k}{\prime}s$).

The second approach is a multivariate approach using multivariate meta-analysis techniques [24] which allows us to also incorporate the estimated covariance between ${\widehat{\beta }}_{1k}$ and ${\widehat{\beta }}_{3k}$ as described above in “Disease progression models” section.

*Performance metrics* We are primarily interested in the bias and coverage of the 95% confidence intervals of the exposure-intercept and exposure-slope effects and also report the empirical SD and the mean of the model based standard errors. For this bias we report both the bias with Monte Carlo Standard Errors (MSE) and also the mean relative bias. We report the joint coverage of the confidence intervals for both the exposure-intercept and exposure-slope effect estimates. The multivariate approach can be used to construct a joint confidence region for the intercept and the slope; such a region will have an elliptic shape, as opposed to a box-shaped region that is obtained by combining the two separate confidence intervals produced by the naïve approach. We also report coverage for a confidence ellipse using the naïve approach by assuming independence (i.e. setting the covariance to be 0).

### Computing

This work was carried out using the computational facilities of the Advanced Computing Research Centre, University of Bristol—http://www.bristol.ac.uk/acrc/. All the simulations and analyses were carried out within STATA 17 and we used the mvmeta package for the multivariate meta-analysis [24]. The R library mixmeta can also be used to perform the multivariate meta-analysis [32]. STATA code used for the simulations and multivariate meta-analysis is available at https://github.com/MLawtonBris/MRtrajectory.

## Results

### Motivating example in Parkinson’s disease

The Discovery cohort analysis is based on 826 individuals with 2,851 observations (average 3.5 ranging from 1 to 7) over an average of 4.2 years follow-up. The average age at diagnosis was 66.0 years (SD 9.6 years) with 538 (65.1%) males. Average disease duration at baseline was 1.2 years (SD 0.9). In a multilevel model where the MDS-UPDRS III was the outcome the fixed effect for the intercept was 23.5 (95% CI: 22.6 to 24.3) and the fixed effect for the linear slope was 2.36 (95% CI: 2.14 to 2.60).Or equivalently, considering the population mean trajectory the predicted outcome at diagnosis was 23.5 and the outcome increases by 2.36 points per year. Average observed BMI at recruitment was 27.4 kg/m^2^ (SD 4.7).

The Tracking cohort is based on 1,517 individuals with 5,024 observations (average 3.3 ranging from 1 to 6) over an average of 3.8 years of follow-up. The average age at diagnosis was 65.9 years (SD 9.2 years) with 987 (65.1%) males which is almost identical to the Discovery cohort. Average disease duration at baseline was 1.3 years (SD 0.9). In a multilevel model where the MDS-UPDRS III was the outcome the fixed effect for the intercept was 20.4 (95% CI: 19.6 to 21.1) and the fixed effect for the linear slope was 2.24 (95% CI: 2.06 to 2.43). Average observed BMI at recruitment was 27.0 kg/m^2^ (SD 4.7).

In Table 1 we report the MR estimates for the effect that BMI has on the intercept and the slope within the two Parkinson’s cohorts and a meta-analysis of the two cohorts. We also report multilevel model (MLM) estimates of the effect that observed BMI at recruitment has on the intercept and slope in the two cohorts and a meta-analysis of the two cohorts. Within the naïve approach to 2SMR the intercept and slope for the two cohorts are meta-analysed separately, each with a standard univariate approach. For the multivariate approach to the MR and also for the MLM estimates the intercept and slope for the two cohorts are meta-analysed jointly with a multivariate approach accounting for the estimated covariance.

**Table 1:** 2SMR analysis on the discovery and tracking cohorts with BMI as the exposure and MDS-UPDRS III^a^ as the outcome

Analysis	Discovery cohort		Tracking cohort		Meta-analysis of two cohorts^b^	
	Intercept	Slope	Intercept	Slope	Intercept	Slope
Naïve MR	− 2.43 (− 7.66, 2.79); *p* = 0.36	0.385 (− 1.01, 1.78); *p* = 0.59	− 0.086 (− 4.41, 4.24); *p* = 0.97	− 0.169 (− 1.28, 0.94); *p* = 0.77	− 1.04 (− 4.37, 2.29); *p* = 0.54	0.045 (− 0.83, 0.92); *p* = 0.92
Multivariate MR	− 2.47 (− 7.69, 2.75); *p* = 0.35	0.389 (− 1.01, 1.79); *p* = 0.59	− 0.065 (− 4.39, 4,26); *p* = 0.98	− 0.165 (− 1.28, 0.95); *p* = 0.77	− 1.04 (− 4.37, 2.29); *p* = 0.54	0.054 (− 0.82, 0.92); *p* = 0.90
Multilevel model estimates	− 0.22 (− 1.08, 0.64); *p* = 0.62	0.303 (0.08, 0.53); *p* = 0.009	0.77 (0.05, 1.50); *p* = 0.037	0.010 (− 0.17, 0.19); *p* = 0.92	0.36 (− 0.19, 0.92); *p* = 0.20	0.127 (− 0.02, 0.27); *p* = 0.081
	Estimated (multivariate approach) correlation^c^ = − 0.52		Estimated (multivariate approach) correlation^c^ = − 0.54		Estimated (multivariate approach) correlation^c^ = − 0.53	

Data shown are estimate (95% CI); *p* value

*MR* Mendelian Randomisation

^a^Note that higher scores on the MDS-UPDRS III are worse symptoms. So, for example, a positive association with the intercept would reflect higher BMI being associated with worse symptoms at baseline

^b^Within the naïve approach to 2SMR the intercept and slope for the two cohorts are meta-analysed separately, each with a standard univariate approach. For the multivariate approach to the 2SMR the intercept and slope for the two cohorts are meta-analysed jointly with a multivariate approach accounting for the estimated covariance

^c^Correlation is reported rather than covariance as it is easier to interpret

From the MLM estimates there is some evidence (*p* = 0.009) that higher BMI is associated with faster progression in the Discovery cohort, where a one SD increase in BMI is associated with an increased slope of 0.30 (95% CI 0.08 to 0.53) points per year. However there is almost no evidence of an effect on the slope in the Tracking cohort (*p* = 0.92) with a smaller estimate of 0.01 (95% CI − 0.17 to 0.19). There is some modest evidence (*p* = 0.037) in the Tracking cohort that higher BMI is associated with higher baseline MDS-UPDRS III at diagnosis where a one SD increase is BMI is associated with an increased intercept of 0.77 (95% CI 0.05 to 1.50) although the effect is in the opposite direction in the Discovery cohort − 0.22 (95% CI − 1.08 to 0.64).

**Fig. 2:**
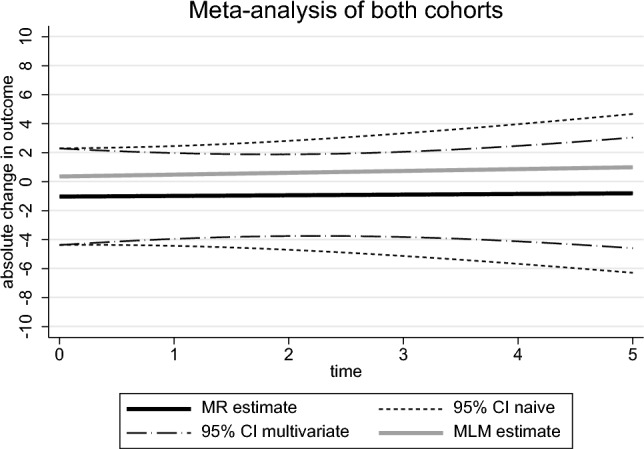
Absolute change in the MDS-UPDRS III for a one point increase in the exposure. Estimated model from the meta-analysis of the two cohorts including the 95% confidence intervals for both the naïve and multivariate Mendelian Randomisation (MR) approach. Also includes the Multilevel Model (MLM) estimate from the meta-analysis of the two cohorts

The MR results from the naïve and multivariate approaches are very similar. There is a large degree of uncertainty in these estimates (especially when compared to MLM estimates) and despite the fact the estimates of the effect of BMI on slope are in opposite directions in the two cohorts the 95% confidence intervals overlap with each other. Given the uncertainty, which is probably due to the limited sample size (for genetic studies) along with the relatively small genetic effects, it is difficult to rule out either a protective or detrimental effect of BMI on disease progression. Figure 2 (meta-analysis) and supplementary Figure 1 (cohort specific effects) clearly shows that when plotting the effect that BMI has on the outcome across time that using the naïve approach (and thus ignoring the covariance) there is a considerable difference to the confidence intervals. When comparing these MR estimates to the MLM estimates the MR estimates have effects that are shifted downwards slightly with slopes closer to the null (0.05 vs. 0.13) and intercept effects that are negative instead of positive (− 1.04 vs. 0.36).

### Simulation study

The results from our simulation with R^2^ = 10% are displayed in Table 2. The results based on the naïve and multivariate approach were almost identical in this simulation, with any differences generally at the third significant figure. The mean bias for the intercept ranged from − 0.006 (MSE: 0.010) in scenario 4 to 0.006 (MSE: 0.011) in scenario 2, while the bias for the slope ranged from − 0.002 (MSE: 0.003) in scenario 4 to 0.003 (MSE: 0.003) in scenario 5. In all six simulation scenarios the bias figures were small and there is little evidence of bias in our simulation study. Accordingly, the mean relative bias was quite small in our simulations, ranging from − 0.2 to 0.3% for the intercept and from − 0.5 to 0.7% for the slope. The coverage of the 95% confidence intervals were also at nominal levels: the coverage for the intercept ranged from 93.4 to 96.1% and for the slope from 94.7 to 95.8%.

**Table 2:** Results from the simulation based on R^2^ of 10%

Scenario (truth)	Correlation^a^ mean (sd)	Intercept—naïve approach					Intercept—multivariate approach				
		Estimate mean (sd)	Mean model se	Coverage 95% CI	Mean relative Bias^b^	Bias^c^ (MSE)	Estimate mean (sd)	Mean model based se	Coverage 95% CI	Mean Relative Bias^b^	Bias^c^ (MSE)
1 (2)	− 0.35 (0.009)	2.00 (0.338)	0.341	94.8%	− 0.2%	− 0.004 (0.011)	2.00 (0.338)	0.341	94.8%	− 0.2%	− 0.004 (0.011)
2 (2)	− 0.38 (0.008)	2.01 (0.349)	0.341	94.7%	0.3%	0.006 (0.011)	2.01 (0.349)	0.341	94.7%	0.3%	0.006 (0.011)
3 (2)	− 0.40 (0.009)	2.00 (0.362)	0.341	93.4%	− 0.1%	− 0.001 (0.011)	2.00 (0.362)	0.341	93.4%	− 0.0%	− 0.001 (0.011)
4 (0)	− 0.40 (0.008)	− 0.01 (0.331)	0.331	95.1%	NA	− 0.006 (0.010)	− 0.01 (0.331)	0.331	95.1%	NA	− 0.006 (0.010)
5 (2)	0.076 (0.010)	2.00 (0.347)	0.341	94.2%	0.1%	0.002 (0.011)	2.00 (0.347)	0.341	94.2%	0.1%	0.002 (0.011)
6 (2)	0.29 (0.009)	2.00 (0.328)	0.341	96.1%	− 0.2%	− 0.004 (0.010)	2.00 (0.328)	0.341	96.1%	− 0.2%	− 0.004 (0.010)

Scenario (truth)	Correlation (sd)	Slope—naïve approach					Slope—multivariate approach				
		Estimate mean (sd)	Mean model based se	Coverage 95% CI	Mean relative Bias^b^	Bias^c^ (MSE)	Estimate mean (sd)	Mean model based se	Coverage 95% CI	Mean relative Bias^b^	Bias^c^ (MSE)
1 (0.45)	− 0.35 (0.009)	0.449 (0.081)	0.083	95.8%	− 0.3%	− 0.001 (0.003)	0.449 (0.081)	0.083	95.8%	− 0.3%	− 0.001 (0.003)
2 (0.25)	− 0.38 (0.008)	0.251 (0.080)	0.081	95.4%	0.3%	0.001 (0.003)	0.251 (0.080)	0.081	95.4%	0.3%	0.001 (0.003)
3 (0)	− 0.40 (0.009)	0.000 (0.079)	0.081	95.3%	NA	0.000 (0.002)	0.000 (0.079)	0.081	95.3%	NA	0.000 (0.002)
4 (0.45)	− 0.40 (0.008)	0.448 (0.081)	0.083	95.3%	− 0.5%	− 0.002 (0.003)	0.448 (0.081)	0.083	95.3%	− 0.5%	− 0.002 (0.003)
5 (0.45)	0.076 (0.010)	0.453 (0.081)	0.083	94.7%	0.7%	0.003 (0.003)	0.453 (0.081)	0.083	94.7%	0.7%	0.003 (0.003)
6 (0.45)	0.29 (0.009)	0.449 (0.083)	0.083	94.7%	− 0.2%	− 0.001 (0.003)	0.449 (0.083)	0.083	94.7%	− 0.2%	− 0.001 (0.003)

NA—relative bias is undefined when the true parameter estimate is zero

*MSE* Monte Carlo Standard Error

^a^Estimated average correlation from the covariance of SNP-intercept and SNP-slope effects. Reported instead of covariance as the correlation is easier to interpret

^b^(average observed estimate-true)/true

^c^Observed estimate—true

The results from our simulation with R^2^ = 2%, displayed in Table 3, were similar. The mean bias for the intercept ranged from − 0.047 (MSE: 0.023) in scenario 4 to 0.030 (MSE: 0.023) in scenario 1, while the bias for the slope ranged from − 0.006 (MSE: 0.006) in scenario 3 to 0.015 (MSE: 0.006) in scenario 5. In all six simulation scenarios the bias figures were small and there is little evidence of bias in our simulation study. Accordingly, the mean relative bias was quite small in our simulations, ranging from − 1.6 to 1.5% for the intercept and − 0.7 to 4.1% for the slope. The coverage of the 95% confidence interval for the intercept ranged from 94.1 to 96.2% and for the slope from 94.5 to 96.0%.

**Table 3:** Results from the simulation based on R^2^ of 2%

Scenario (truth)	Correlation^a^ mean (sd)	Intercept—naïve approach					Intercept—multivariate approach				
		Estimate mean (sd)	Mean model se	Coverage 95% CI	Mean relative Bias^b^	Bias^c^ (MSE)	Estimate mean (sd)	Mean model based se	Coverage 95% CI	Mean relative Bias^b^	Bias^c^ (MSE)
1 (2)	− 0.35 (0.009)	2.03 (0.717)	0.764	96.2%	1.5%	0.030 (0.023)	2.03 (0.717)	0.764	96.2%	1.5%	0.030 (0.023)
2 (2)	− 0.38 (0.008)	1.97 (0.778)	0.764	94.1%	− 1.6%	− 0.033 (0.025)	1.97 (0.778)	0.764	94.1%	− 1.6%	− 0.033 (0.025)
3 (2)	− 0.40 (0.008)	2.02 (0.789)	0.764	94.3%	1.2%	0.025 (0.025)	2.03 (0.789)	0.764	94.3%	1.3%	0.025 (0.025)
4 (0)	− 0.40 (0.008)	− 0.05 (0.739)	0.741	95.1%	NA	− 0.047 (0.023)	− 0.05 (0.739)	0.741	95.1%	NA	− 0.047 (0.023)
5 (2)	0.077 (0.010)	2.00 (0.768)	0.764	95.0%	0.1%	0.003 (0.024)	2.00 (0.768)	0.764	95.0%	0.1%	0.003 (0.024)
6 (2)	0.29 (0.009)	2.02 (0.769)	0.764	95.2%	0.8%	0.016 (0.024)	2.02 (0.769)	0.764	95.2%	0.8%	0.016 (0.024)

Scenario	Correlation (sd)	Slope—naïve approach					Slope—multivariate approach				
		Estimate mean (sd)	Mean model based se	Coverage 95% CI	Mean relative Bias^b^	Bias^c^ (MSE)	Estimate mean (sd)	Mean model based se	Coverage 95% CI	Mean relative Bias^b^	Bias^c^ (MSE)
1 (0.45)	− 0.35 (0.009)	0.447 (0.181)	0.185	95.7%	− 0.7%	− 0.003 (0.006)	0.447 (0.181)	0.185	95.7%	− 0.7%	− 0.003 (0.006)
2 (0.25)	− 0.38 (0.008)	0.260 (0.182)	0.181	95.1%	4.1%	0.010 (0.006)	0.260 (0.182)	0.181	95.1%	4.1%	0.010 (0.006)
3 (0)	− 0.40 (0.008)	− 0.006 (0.181)	0.180	94.5%	NA	− 0.006 (0.006)	− 0.006 (0.181)	0.180	94.5%	NA	− 0.006 (0.006)
4 (0.45)	− 0.40 (0.008)	0.463 (0.185)	0.185	95.2%	2.9%	0.013 (0.006)	0.463 (0.185)	0.185	95.2%	2.9%	0.013 (0.006)
5 (0.45)	0.077 (0.010)	0.465 (0.188)	0.185	95.0%	3.3%	0.015 (0.006)	0.465 (0.188)	0.185	95.0%	3.3%	0.015 (0.006)
6 (0.45)	0.29 (0.009)	0.449 (0.178)	0.185	96.0%	− 0.3%	− 0.001 (0.006)	0.449 (0.178)	0.185	96.0%	− 0.3%	− 0.001 (0.006)

NA—mean relative bias is undefined when the true parameter estimate is zero

*MSE* Monte Carlo Standard Error

^a^Estimated correlation from the covariance of SNP-intercept and SNP-slope effects Reported instead of covariance as the correlation is easier to interpret

^b^(average observed estimate-true)/true

^c^Observed estimate—true

Table 4 shows the joint coverage for the different approaches. For the naïve approach the joint coverage of the confidence rectangle ranged from 89.0 to 91.5% when the R^2^ was 10% and from 89.9 to 92.5% when the R^2^ was 2%. This is not surprising, as the joint coverage of two separate 95% confidence intervals is approximately 0.95^2^ = 0.9025. The joint coverage of the naïve confidence ellipse (assuming covariance = 0) ranged from 93.5 to 95.5% when the R^2^ was 10% and from 93.7 to 95.9% when the R^2^ was 2%.Using the multivariate approach the joint coverage for the confidence ellipses ranged from 94.6 to 95.2% when the R^2^ was 10% and from 93.3 to 96.1% when the R^2^ was 2%.

**Table 4:** Joint coverage of the intercept and slope

Scenario	R^2^ of 10%			R^2^ of 2%		
	Coverage of naïve confidence rectangle (%)	Coverage of naïve confidence ellipse (%)	Coverage of multivariate confidence ellipse (%)	Coverage of naïve confidence rectangle (%)	Coverage of naïve confidence ellipse (%)	Coverage of multivariate confidence ellipse (%)
1	91.5	95.2	94.9	92.5	95.9	95.5
2	90.6	94.1	94.9	89.9	94.1	93.3
3	89.6	93.5	94.7	90.0	94.2	95.4
4	90.8	95.3	94.9	91.0	93.7	96.1
5	89.0	94.4	94.6	90.5	94.8	94.5
6	91.3	95.5	95.2	91.5	94.6	95.8

For the naïve approach we used two approaches: a confidence rectangle using the two confidence intervals and a confidence ellipse where the covariance is assumed to be zero. Whilst for the multivariate approach we have drawn a confidence ellipse using the estimated covariance All of our confidence regions are estimated at the 95% level

Supplementary table 1, shows the estimates of the areas for the confidence ellipses using the naïve and multivariate approaches. In all scenarios (except scenario 5 where the correlation is small) the average area of the confidence ellipses is smaller using the multivariate approach. Both the naïve and multivariate confidence ellipses have coverage close to the nominal 95% level and in this situation the confidence region with the smaller area, the multivariate approach, would be preferred.

In our sensitivity analysis, see supplementary table 2, with a reduced sample size and unbalanced data the naïve and multivariate approach still gave near identical results. Not surprisingly the uncertainty in the estimates is much larger than observed in Table 2 and 3. The bias was small roughly − 0.003 (MSE 0.049) for the intercept and − 0.006 (MSE 0.014) for the slope and accordingly the mean relative bias was small—approximately − 0.15% for the intercept and approximately − 1.35% for the slope. The coverage of the 95% CI was 94.8% and 95.0% for the intercept (using the naïve and multivariate approach respectively) and 94.4% and 94.3% for the slope (using the naïve and multivariate approach respectively). The joint coverage for the sensitivity analysis was 90.7% for the naïve approach, 93% for the naïve ellipse and 93.8% for the multivariate ellipse. The average difference in area for these two ellipses is very striking in this sensitivity analysis (supplementary table 1) even though the coverage is very similar.

## Discussion

Our simulations show that 2SMR gives unbiased estimates with good coverage when the outcome is generated as a linear trajectory, with intercept and slope caused by the exposure. If researchers are interested in graphs of the effect that an exposure has on an outcome over time then a multivariate meta-analysis method that allows the estimation of a covariance term could give substantially different confidence intervals. The multivariate meta-analysis method also provides appropriate joint coverage of both the intercept and slope effects by drawing a parsimonious confidence ellipse which could also provide a joint hypothesis test. However, rate of progression is often of greater relevance for individuals who have recently been diagnosed with a disease. We have shown that a naïve approach will give almost identical estimates to a multivariate approach when the slope is the focus of interest.

In our simulations and this example we assumed that the exposure that did not vary over time, but in the case of BMI this is an over-simplification. It is also important to note that MR estimates the lifetime effect of an exposure on an outcome. It has been shown previously that the effect of the fat mass and obesity-associated gene (FTO) has an effect on BMI that is not constant with age [33, 34]. Hence the effect that BMI has on an outcome may depend on age. Methods have been proposed that use time-varying SNP-exposure estimates [34] or multivariable MR [35] to account for this time-varying nature of the exposure.

Mendelian Randomisation is dependent on the three instrumental variable assumptions which are required for a valid hypothesis test of a causal effect between exposure and outcome [36]. We also make the assumption that there is a linear association between the exposure and the outcome (in this case the intercept and slope) and that this effect is homogenous [36, 37]. This homogeneity assumption means there should be no effect modifiers in the confounders of the exposure and outcome. If these assumptions are not met then bias would be unpredictable. An alternative no simultaneous heterogeneity (NOSH) assumption has been proposed that allows for heterogeneity in the exposure outcome effect as long as this heterogeneity is independent of the IV and any heterogeneity in the IV exposure association [38]. A further assumption is of monotonicity where the direction of the IV exposure effect is the same for all individuals [36].

In some situations progression trajectories might be non-linear over time [9] and our multivariate meta-analysis approach could be adapted to have multiple time terms using fractional polynomials [9] or splines [39]. In such an example we would recommend deriving the difference in trajectories per additional copy of the effect allele at some time-point. The chosen time-point will depend on both the length of follow-up in the data and knowledge of a clinically meaningful time in that particular disease. We would also recommend plotting a non-linear graph similar to that displayed in Fig. 2. Non-linearity could also be a problem in our applied example, but the low number of observations we have per-person (on average) is limited so we had insufficient data to test and account for non-linear trajectories.

The models we have used assume there is a residual for each observed measure of the outcome (${\varepsilon }_{ij}$ in Eq. 3) where these residuals are independent and with constant variance. Multilevel models can be modified to allow for autocorrelation or complex measurement error where the variance of the level 1 residuals changes over time [9]. If there is an underlying factor affecting measurement error in all measures for an individual—e.g. if individuals with impaired hearing tend to always score lower due to difficulties completing the questionnaire—then the measurement errors for all the measures will be correlated. However, this correlation will be subsumed into the effect of the “latent progression” factor, and thus also included in the model-based estimate of correlation between intercept and slope. This type of measurement error would not bias the MR in this example, because hearing loss would be essentially a confounder of different measures of outcome [40].

Another potential issue in observational longitudinal data occurs when the follow-up data is missing not at random (informative drop-out). An example is when an individual’s disability progresses such that clinic visit attendance becomes impossible which could bias trajectories towards the null. This could be explored using pattern-mixture models, selection models or other similar approaches [41–44] that under some untestable assumptions would be less biased than standard multilevel models. We have previously used pattern-mixture models to look at disease progression in PD which gave us similar results to a standard multilevel model providing some evidence that informative drop-out did not bias our results [8].

Allowing an exposure to affect both the intercept and slope is possibly an over-simplistic way to model progression in PD. In reality there should be a true time zero where the individual has no impairment caused by the disease. However in PD there is a long prodromal (prediagnostic) period [45] and by the time of diagnosis there is already considerable motor impairment and large variability in motor impairment between individuals [46]. Modelling the effect of the exposure on the intercept using disease duration from diagnosis as the time-axis reflects some period of prodromal progression. The current progression data that is available for most cohorts of individuals with PD does not allow us to observe this prodromal period.

There is some evidence for BMI being a risk factor for PD [2] hence our results may be affected by index event bias. We would expect confounders to act in the same direction for both incidence of PD and progression. That is confounders that increase risk of PD would also be expected to increase its severity/progression. This would result in a negative index event bias and thus if higher BMI reduced severity at baseline and progression we would expect that MR effect to be shifted upwards in this study. This could be one explanation for the lack of causal of BMI on PD progression in our study. Other explanations include that there is in truth no causal effect of BMI on PD progression, chance, lack of power or selection bias due to competing risks. A negative effect of BMI on the intercept such as in our MR estimate (albeit with a high *p* value) would be in agreement with a previous meta-analysis showing that higher BMI was associated with lower disease severity [18].

A 2SMR package in STATA [47] by default forces the residual variance to be 1 when residual variance is less than one or freely estimates the residual variance when it is greater than one, thus allowing for overdispersion but not underdispersion. Not allowing for underdispersion is intuitive since we are weighting by the inverse of the variance of each SNP and it would be strange to allow for an estimator that is more precise than the variability in our SNP-outcome measures. Our multivariate meta-analysis approach will not allow for overdispersion so is equivalent to forcing the residual variance to be 1. If there was overdispersion then our method could provide an estimate that is overly precise. However, it is possible to test and allow for overdispersion in a structural equation model framework by first weighting the SNP-outcome and SNP-exposure estimates by the Cholesky decomposition of the inverse of SNP-outcome covariance matrices. This approach is described, along with STATA code, in the following article (Sect. 5.1) [48].

Our approach is very similar to a two-stage multivariate MR method for mixed outcomes (called MRMO) [49] which studies the effect of an exposure on multiple outcomes (which could be mixed such as binary and continuous). MRMO also describes a procedure to estimate a *p *value for whether the exposure influences any of the outcomes by using a Wald test. We could also estimate a Wald test using our estimates for the exposure-intercept and exposure-slope along with their covariance matrix. This would be a joint hypothesis test for whether the exposure-intercept is zero and the exposure-slope is zero and could be interpreted as whether the exposure has any effect on progression. In this paper we have focussed on confidence intervals and regions rather than *p* values. Other researchers have argued that with time-varying exposures it is difficult to define a causal effect or that the assumptions behind those causal effects are questionable. In these situations where the definition of an effect size is debatable MR (with valid instruments) can be used to test the null hypothesis that exposure at any time does not cause the outcome [33, 50].

Using our multivariate meta-analysis method it would be possible to carry out the MR-Egger approach by also estimating an intercept term allowing us to correct for directional pleiotropy. In future work we will explore whether other methods such as the median and mode approaches could be adapted for this purpose. Given the large uncertainty from the analysis presented here, large consortiums with many longitudinal PD cohorts will be required to have sufficient power to detect whether exposures are related to progression.

This paper shows that it is possible to use multivariate meta-analysis to carry out two sample Mendelian Randomisation when using an outcome that is repeatedly measured over time. The associations between an exposure and the intercept and linear slope of the repeatedly measured trait are unbiased with valid confidence intervals. These methods enable researchers to use two sample Mendelian Randomisation to test hypotheses about exposures causing disease progression in neurodegenerative diseases and other situations with longitudinal outcome data.

### Supplementary Information

Below is the link to the electronic supplementary material.

Supplementary file1 (DOCX 102 kb)

## Data Availability

Data from the Oxford Discovery cohort is available on request from https://www.dpag.ox.ac.uk/opdc/research/external-collaborations. Data from the Tracking Parkinsons cohort is available on request from https://www.trackingparkinsons.org.uk/about-1/data/.
