# Supplementary material for: Two sample Mendelian Randomisation using an outcome from a multilevel model of disease progression
Source: Eur J Epidemiol. 2024 Jan 28;39(5):521–33. doi: 10.1007/s10654-023-01093-2 (PMC11219432; doi:10.1007/s10654-023-01093-2)
Supplement: Supplementary file 1 — Supplementary file1 (DOCX 102 kb) [file 10654_2023_1093_MOESM1_ESM.docx]

Supplementary table 1. Estimated areas from the confidence ellipses using the naïve (covariance=0) and the multivariate approach along with the model estimated correlation (between exposure-intercept and exposure-slope estimates) used to construct the multivariate confidence ellipse. Data is mean (standard deviation).

|  | R^2^ of 10% | | | R^2^ of 2% | | |
| --- | --- | --- | --- | --- | --- | --- |
| Scenario | Naïve approach area | Multivariate approach area | Multivariate approach correlation^a^ | Naïve approach area | Multivariate approach area | Multivariate approach correlation^a^ |
| 1 | 0.531 (0.006) | 0.498 (0.005) | -0.35 (0.009) | 2.66 (0.03) | 2.50 (0.03) | -0.35 (0.009) |
| 2 | 0.521 (0.005) | 0.481 (0.005) | -0.38 (0.008) | 2.61 (0.03) | 2.41 (0.02) | -0.38 (0.008) |
| 3 | 0.518 (0.006) | 0.475 (0.005) | -0.40 (0.009) | 2.60 (0.03) | 2.38 (0.02) | -0.40 (0.008) |
| 4 | 0.516 (0.006) | 0.472 (0.005) | -0.40 (0.008) | 2.58 (0.03) | 2.37 (0.02) | -0.40 (0.008) |
| 5 | 0.531 (0.005) | 0.530 (0.005) | 0.076 (0.010) | 2.66 (0.03) | 2.65 (0.03) | 0.077 (0.010) |
| 6 | 0.532 (0.006) | 0.509 (0.005) | 0.29 (0.009) | 2.66 (0.03) | 2.55 (0.03) | 0.29 (0.009) |
| Sensitivity analysis | 12.7 (0.6) | 9.6 (0.4) | -0.65 (0.019) |  |  |  |

^a^Correlation is reported instead of covariance as it is easier to interpret.

Supplementary Table 2. Results from the simulation with a reduced sample size and unbalanced data (between 1-4 observations per person)

|  |  | Intercept – naïve approach | | | | | Intercept – multivariate approach | | | | |
| --- | --- | --- | --- | --- | --- | --- | --- | --- | --- | --- | --- |
| Scenario (truth) | Correlation^a^ mean (sd) | Estimate mean (sd) | Mean model se | Coverage 95% CI | Mean relative Bias^b^ | Bias^c^  (MSE) | Estimate mean (sd) | Mean model based se | Coverage 95% CI | Mean relative Bias^b^ | Bias^c^  (MSE) |
| 1 (2) | -0.65 (0.019) | 2.00 (1.54) | 1.52 | 94.8% | -0.2% | -0.003  (0.049) | 2.00 (1.54) | 1.52 | 95.0% | -0.1% | -0.002  (0.049) |
|  |  | Slope – naïve approach | | | | | Slope – multivariate approach | | | | |
| Scenario | Correlation (sd) | Estimate mean (sd) | Mean model based se | Coverage 95% CI | Mean relative Bias^b^ | Bias^c^  (MSE) | Estimate mean (sd) | Mean model based se | Coverage 95% CI | Mean relative Bias^b^ | Bias^c^  (MSE) |
| 1 (0.45) | -0.65 (0.019) | 0.444 (0.454) | 0.446 | 94.4% | -1.4% | -0.006  (0.014) | 0.444 (0.454) | 0.446 | 94.3% | -1.3% | -0.006  (0.014) |
|  | | Joint coverage – naïve approach | | | | | Joint coverage – multivariate approach | | | | |
|  | | Rectangle – 90.7% ; Ellipse 93% | | | | | 93.8% | | | | |

^a^Estimated correlation from the covariance of SNP-intercept and SNP-slope effects

^b^(observed estimate-true)/true

^c^ observed estimate - true

MSE = Monte Carlo Standard Error

Supplementary Figure 1. Absolute change in the MDS-UPDRS III for a one point increase in the exposure. 1a shows the estimated model from the Oxford Discovery cohort including the 95% confidence intervals for both the naïve and multivariate Mendelian Randomisation (MR) approach and 1b the same from the Tracking cohort. Also includes the Multilevel Model (MLM) estimates.

|  |  |
| --- | --- |
